# Interobserver agreement on definition of the target volume in stereotactic radiotherapy for pancreatic adenocarcinoma using different imaging modalities

**DOI:** 10.1007/s00066-023-02085-7

**Published:** 2023-06-02

**Authors:** E. Gkika, D. Kostyszyn, T. Fechter, C. Moustakis, F. Ernst, J. Boda-Heggemann, G. Sarria, K. Dieckmann, S. Dobiasch, M. N. Duma, F. Eberle, K. Kroeger, B. Häussler, V. Izaguirre, D. Jazmati, S. Lautenschläger, F. Lohaus, F. Mantel, J. Menzel, S. Pachmann, M. Pavic, K. Radlanski, O. Riesterer, S. Gerum, F. Röder, J. Willner, S. Barczyk, D. Imhoff, O. Blanck, A. Wittig, M. Guckenberger, Anca-L. Grosu, T. B. Brunner

**Affiliations:** 1 Department of Radiation Oncology, University Medical Center Freiburg, Robert Koch Str 3, Freiburg, Germany; 2 Department of Radiation Oncology, University Medical Center Muenster, Muenster, Germany; 3 Institute for Robotics and Cognitive Systems, University of Luebeck, Luebeck, Germany; 4 Department of Radiation Oncology, Faculty of Medicine Mannheim, Department of Radiation Oncology, University of Heidelberg, Mannheim, Germany; 5 Department of Radiation Oncology, University Departments of the MedUni Vienna, Vienna General Hospital, Vienna, Austria; 6 Department of Radiation Oncology, Klinikum Rechts der Isar, TU Munich, Munich, Germany; 7 Department of Radiotherapy and Radiation Oncology, University Hospital Jena, Friedrich-Schiller University, Jena, Germany; 8 Department of Radiation Oncology, University Hospital Marburg, Marburg, Germany; 9 Department of Radiation Oncology, University Hospital Bonn, Bonn, Germany; 10 Radiation Oncology Dr. Häussler/Dr. Schorer, Munich, Germany; 11 Department of Radiation Oncology, University Hospital Halle, Halle, Germany; 12 Proton Therapy Centre, University Hospital Essen, Essen, Germany; 13 Department of Radiation Oncology, University Hospital, Marburg, Germany; 14 Department of Radiation Oncology, University Hospital Dresden, Dresden, Germany; 15 Department of Radiation Oncology, University Hospital Würzburg, Würzburg, Germany; 16 Department of Radiation Oncology, University Hospital Hannover, Hannover, Germany; 17 Department of Radiation Oncology, Weilheim Clinic, Weilheim, Germany; 18 Department of Radiation Oncology, University Hospital Zurich, University of Zurich, Zurich, Switzerland; 19 Department of Radiation Oncology, Charite, University Hospital Berlin, Berlin, Germany; 20 Centre for Radiation Oncology KSA-KSB, Kantonsspital Aarau, Aarau, Switzerland; 21 Department of Radiation Oncology, University Clinic, Paracelsus Medical University (PMU), Salzburg, Austria; 22 Department of Radiation Oncology, University Hospital Bayreuth, Bayreuth, Germany; 23 Center for Radiation Oncology, Belegklinik am St. Agnes—Hospital, Bocholt, Germany; 24 Department of Radiation Oncology, Saphir Radiosurgery, University Hospital Frankfurt, Frankfurt, Germany; 25 Saphir Radiosurgery, University Hospital Schleswig-Holstein, Campus Kiel, Kiel, Germany; 26 Department of Therapeutic Radiology and Oncology, Comprehensive Cancer Center, Medical University of Graz, Graz, Austria

**Keywords:** Interobserver agreement, Target volume definition, Stereotactic radiotherapy, Pancreatic cancer, Imaging

## Abstract

**Purpose:**

The aim of this study was to evaluate interobserver agreement (IOA) on target volume definition for pancreatic cancer (PACA) within the Radiosurgery and Stereotactic Radiotherapy Working Group of the German Society of Radiation Oncology (DEGRO) and to identify the influence of imaging modalities on the definition of the target volumes.

**Methods:**

Two cases of locally advanced PACA and one local recurrence were selected from a large SBRT database. Delineation was based on either a planning 4D CT with or without (w/wo) IV contrast, w/wo PET/CT, and w/wo diagnostic MRI. Novel compared to other studies, a combination of four metrics was used to integrate several aspects of target volume segmentation: the Dice coefficient (DSC), the Hausdorff distance (HD), the probabilistic distance (PBD), and the volumetric similarity (VS).

**Results:**

For all three GTVs, the median DSC was 0.75 (range 0.17–0.95), the median HD 15 (range 3.22–67.11) mm, the median PBD 0.33 (range 0.06–4.86), and the median VS was 0.88 (range 0.31–1). For ITVs and PTVs the results were similar. When comparing the imaging modalities for delineation, the best agreement for the GTV was achieved using PET/CT, and for the ITV and PTV using 4D PET/CT, in treatment position with abdominal compression.

**Conclusion:**

Overall, there was good GTV agreement (DSC). Combined metrics appeared to allow a more valid detection of interobserver variation. For SBRT, either 4D PET/CT or 3D PET/CT in treatment position with abdominal compression leads to better agreement and should be considered as a very useful imaging modality for the definition of treatment volumes in pancreatic SBRT. Contouring does not appear to be the weakest link in the treatment planning chain of SBRT for PACA.

## Introduction

Exact definition of the target volume plays a crucial role in radiotherapy, especially in stereotactic body radiotherapy (SBRT), due to the high doses applied, the steep dose gradients, and the small margins. In pancreatic cancer, treatment intensification with higher doses might play a significant role, but on the other hand, the vicinity to organs at risk such as the duodenum or the stomach poses a significant complexity in accurately delineating the target volumes. Inconsistencies in the delineation might lead to recurrences or higher exposure of organs at risk and subsequently to a higher risk for toxicities. Several attempts have been made to reduce exposure of organs at risk, either by reducing the dose or using a simultaneous integrated protection volume [1, 2]. However, appropriate planning hinges on accurate definition of the target and the organs at risk.

The aim of this study within the Radiosurgery and Stereotactic Radiotherapy Working Group of the German Society of Radiation Oncology (DEGRO) was to evaluate the interobserver variability concerning the definition of gross tumor volumes (GTVs), internal target volumes (ITVs), and planning target volumes (PTVs) in three patients using different metrics in a group of experts. Furthermore, we aimed to analyze the challenges concerning definition of the target volume. This is a prerequisite to initiating a harmonizing process within our group in order to reduce interobserver variabilities and standardize target volume definition. Moreover, we aimed at identifying organ-specific imaging modalities which are better suited for definition of the target volumes (GTV, ITV, and PTV) for pancreatic cancer to ensure the desired quality for SBRT as previously discussed within the DEGRO Working Group Radiosurgery and Stereotactic Radiotherapy and the DGMP Working Group for Physics and Technology in Stereotactic Radiotherapy [3].

## Materials and methods

After approval from the leading ethics committee of the medical faculty of the University of Kiel (reference number KI D 514/18), the SBRT pancreatic databases of the lead institutions were screened for suitable cases for this study. The aim was to select different clinical scenarios with a certain complexity depicting typical SBRT indications using different imaging modalities, in order to depict which imaging modality leads to better interobserver agreement and is thus better suited for target volume definition. Finally, three patients with histologically proven pancreatic adenocarcinoma treated with SBRT were selected for the evaluation of interobserver variability concerning definition of the GTV, ITV, and PTV:

1. The first patient was diagnosed with a local recurrence after initial pancreaticoduodenectomy and adjuvant chemotherapy. For definition of the treatment volume, experts were provided with a pretreatment 4D planning CT with abdominal compression in treatment position using an individualized vacuum cushion, an additional planning FDG-PET/CT with abdominal compression, and a diagnostic CT with IV contrast.
2. The second patient had locally advanced unresectable pancreatic adenocarcinoma of the head of the pancreas, which progressed under initial chemotherapy. Experts were provided with a 4D planning PET/CT with abdominal compression in treatment position using an individualized vacuum cushion without IV contrast and an additional diagnostic MRI.
3. The third patient also had locally advanced unresectable pancreatic adenocarcinoma of the pancreatic head, which progressed under initial chemotherapy. Experts were provided with a 4D planning CT in treatment position with oral and intravenous (IV) contrast agent.

Anonymized datasets were sent to group members with pancreatic SBRT experience amongst an international group of experts from Germany, Austria, and Switzerland within the stereotactic radiotherapy group of the DEGRO. The number of cases was chosen to have enough statistical power, as previously described [4, 5].

For the analytic comparison of the GTV, ITV, and PTV, we used different qualitative and geometric metrics as described by Taha and Hanbury [6]:

A. The Dice–Sorensen coefficient (DSC), an overlap-based evaluation, which is the most used metric in medical image segmentation.
B. The symmetric Hausdorff distance (HD) is a spatial distance-based metric used as an outlier-sensitive evaluation that ensures exact contours and alignment.
C. The probabilistic distance (PBD) is a probabilistic metric that strongly penalizes alignment errors (when the segmented size of the volume is correct but the overlap is low). It is relevant when the alignment is of more interest than volume or contour.
D. The volumetric similarity (VS), a volume-based metric, only compares the volume of two segmentations, assuming that the alignment is optimal. The overlap is not considered.

True negatives were not considered by any of the metrics and, hence, the number of background voxels did not influence the evaluation significantly. For DSC (range [0–1]) and VS (range [0–1]), higher values show a better interobserver agreement (IOA), while for HD (range [0–∞]) and PBD (range [−1–∞]), lower values correlate with a better IOA.

For the analysis a Python3 framework was created to compute the metrics automatically, which was used to conduct the experiments for each dataset. All comparisons were performed between all experts.

## Results

A total of 24 experts participated in the study and 19 structure sets per case were sent for evaluation. Not all experts provided structure sets for every case. For all GTVs the median DSC for all cases was 0.75 (range 0.17–0.95), the median HD 15 (range 3.22–67.11) mm, the median PBD 0.33 (range 0.06–4.86), and the median VS was 0.88 (range 0.31–1). The results were also similar for ITV and PTV, as shown in Table 1, but there were some differences between the imaging modalities concerning the definition of the treatment volumes.

**Table 1:** Interobserver variability for all patients concerning the definition of the target volumes

IOA	DSC overlap-based	HD Distance-based	PBD Probabilistic	VS Volume-based
**Gross tumor volume**				
*All patients*				
Minimum	0.17	3.22	0.06	0.34
Maximum	0.95	67.11	4.86	1.0
Mean	0.72	17.1	0.51	0.85
Median	0.75	15	0.33	0.88
Std	0.14	9	0.68	0.12
*Patient 1(4D planning CT without IV contrast, planning PET/CT, diagnostic CT with IV contrast)*				
Minimum	0.56	3.22	0.06	0.58
Maximum	0.95	27.24	0.79	1.0
Mean	0.77	11.43	0.31	0.87
Median	**0.78**	**10.35**	**0.28**	0.88
Std	0.07	5.32	0.13	0.09
*Patient 2 (4D planning PET/CT without IV contrast, diagnostic MRI)*				
Minimum	0.45	6.6	0.08	0.48
Maximum	0.92	30.78	1.2	1.0
Mean	0.74	16.5	0.36	0.88
Median	0.7	15.4	0.31	**0.91**
Std	0.08	5.37	0.18	0.11
*Patient 3 (4D planning CT with IV contrast, diagnostic CT with IV contrast)*				
Minimum	0.17	8.73	0.17	0.34
Maximum	0.85	67.11	4.86	1.0
Mean	0.64	23.54	0.87	0.81
Median	0.73	21.61	0.38	0.84
Std	0.2	10.57	1.07	0.14
**Internal tumor volume**				
*All patients*				
Minimum	0.2	3.87	0.06	0.28
Maximum	0.95	70.5	4.01	1.0
Mean	0.69	18.21	0.56	0.78
Median	0.72	15.87	0.39	0.82
Std	0.14	9.42	0.52	0.16
*Patient 1 (4D planning CT without IV contrast, diagnostic PET/CT, diagnostic CT with IV contrast)*				
Minimum	0.39	3.87	0.06	0.39
Maximum	0.95	722.56	1.59	1.0
Mean	0.71	12.55	0.45	0.77
Median	0.73	**12.18**	0.37	0.80
Std	0.11	4.03	0.28	0.15
*Patient 2 (4D planning PET/CT without IV contrast, diagnostic MRI)*				
Minimum	0.52	6.67	0.12	0.53
Maximum	0.89	34.07	0.94	1.0
Mean	0.76	15.73	0.33	0.84
Median	**0.77**	15.03	**0.3**	**0.86**
Std	0.08	5.42	0.16	0.12
*Patient 3 (4D planning CT with IV contrast, diagnostic CT with IV contrast)*				
Minimum	0.2	11.09	0.24	0.28
Maximum	0.8	70.56	4.01	1.0
Mean	0.58	26.67	0.92	0.72
Median	0.6	23.64	0.66	0.72
Std	0.16	10.94	0.73	0.18
**Planning target volume**				
*All patients*				
Minimum	0.26	3.87	0.04	0.37
Maximum	0.96	70.56	2.88	1.0
Mean	0.74	18.53	0.41	0.82
Median	0.77	15.9	0.30	0.84
Std	0.13	9.15	0.537	0.13
*Patient 1 (4D planning CT without IV contrast, planning 3D PET/CT, diagnostic CT with IV contrast)*				
Minimum	0.44	3.87	0.04	0.44
Maximum	0.96	30.24	1.26	1.0
Mean	0.76	14.43	0.35	0.80
Median	0.77	**13.16**	0.29	0.82
Std	0.10	6.01	0.22	0.13
*Patient 2 (4D planning PET/CT without IV contrast, diagnostic MRI)*				
Minimum	0.61	6.9	0.09	0.62
Maximum	0.91	33.17	0.63	1.0
Mean	0.81	15.11	0.25	0.88
Median	**0.81**	14.2	**0.24**	**0.89**
Std	0.06	4.75	0.10	0.09
*Patient 3 (4D planning CT with IV contrast, diagnostic CT with IV contrast)*				
Minimum	0.26	10.3	0.18	0.37
Maximum	0.85	70.56	2.88	1.0
Mean	0.65	26.05	0.65	0.77
Median	0.69	24.0	0.45	0.79
Std	0.14	10.35	0.51	0.15

Best results achieved are marked **bold**

*IOA* interobserver agreement, *Std* standard deviation, *DSC* Dice–Sorensen coefficient, *HD* symmetric Hausdorff distance, *PBD* probabilistic distance, *VS* volumetric similarity

**Fig. 1:**
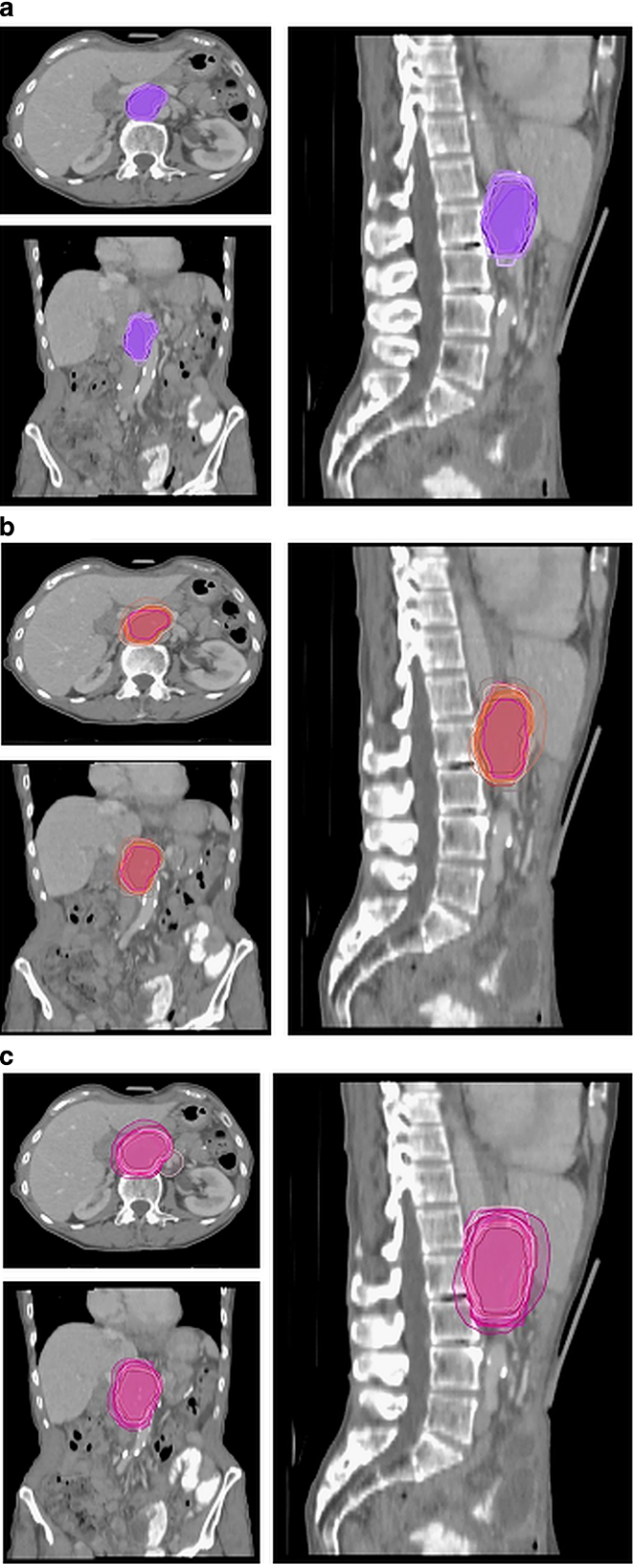
Case 1: patient with relapse after pancreatoduodenectomy. Definition of the target volumes performed on the basis of 4D CT in combination with a planning 3D PET/CT and a diagnostic CT with IV contrast. **a** GTV, **b** ITV, **c** PTV. *GTV* gross tumor volume, *ITV* internal target volume, *PTV* planning target volume

In the first case, the patient was treated with SBRT due to a relapse. The definition of the target volumes was based on 4D CT in combination with a 3D planning PET/CT with abdominal compression and a diagnostic CT with IV contrast (Fig. 1) without a diagnostic MRI. The median GTV was 31.75 (range 21.5–59.2) ml, the median ITV was 54.1 (range 27.6–62.4) ml, and the median PTV 97.65 (range 60.7–214.4) ml. For the GTVs the median DSC was 0.78, the median HD 10.35, the median PBD 0.28, and the median VS was 0.88, and for the ITVs the median DSC was 0.73, the median HD 12.18, the median PBD 0.37, and the median VS was 0.80.

**Fig. 2:**
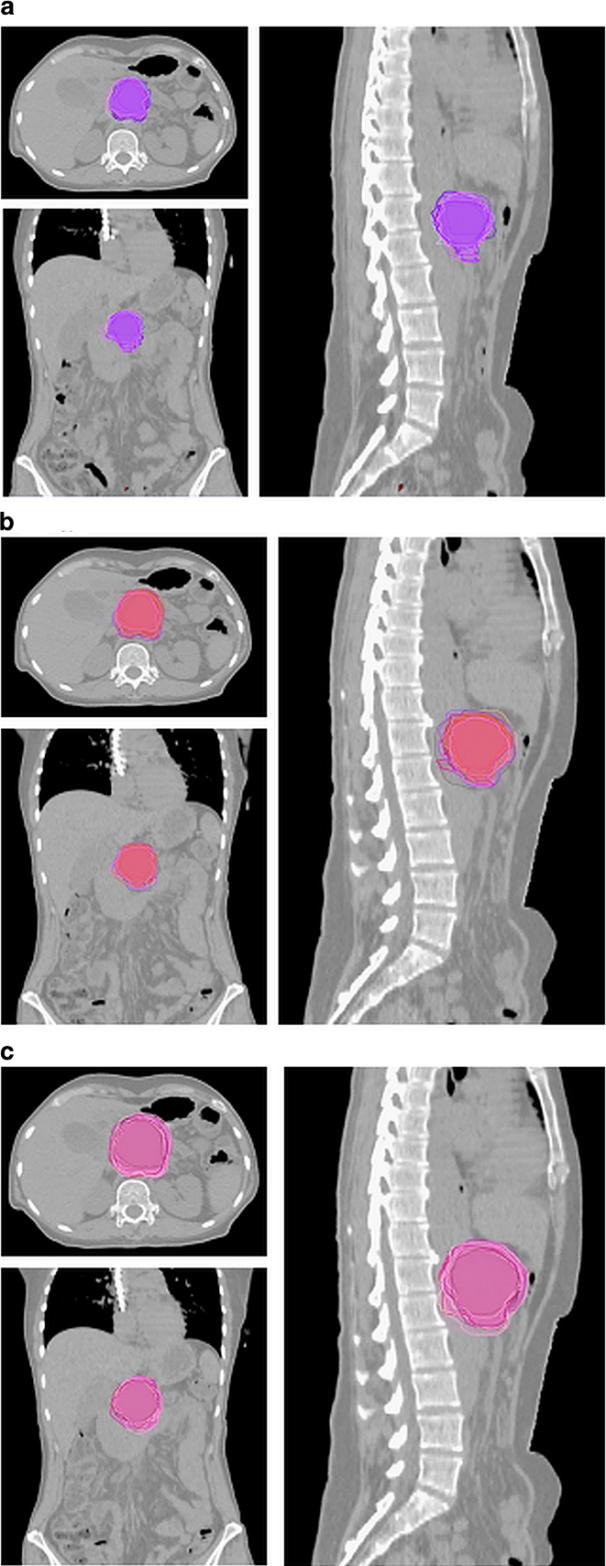
Case 2: patient with progression after initial chemotherapy. Definition of the target volumes based on 4D PET/CT in combination with diagnostic MRI with IV contrast. **a** GTV, **b** ITV, **c** PTV

In the second case, the patient had locally advanced (unresectable) pancreatic cancer and was irradiated due to progression under chemotherapy. The definition of the target volumes was performed on the basis of 4D PET/CT with abdominal compression, in combination with a diagnostic MRI with IV contrast (Fig. 2). The median GTV was 45.3 (range 22.3–73.6) ml, the median ITV was 73.4 (range 51.3–145.4) ml, and the median PTV 138.65 (range 95.3–215.5) ml. For the GTVs the median DSC was 0.7, the median HD 15.4, the median PBD 0.31, and the median VS was 0.91, and for the ITVs the median DSC was 0.77, the median HD 15.03, the median PBD 0.3, and the median VS was 0.86.

**Fig. 3:**
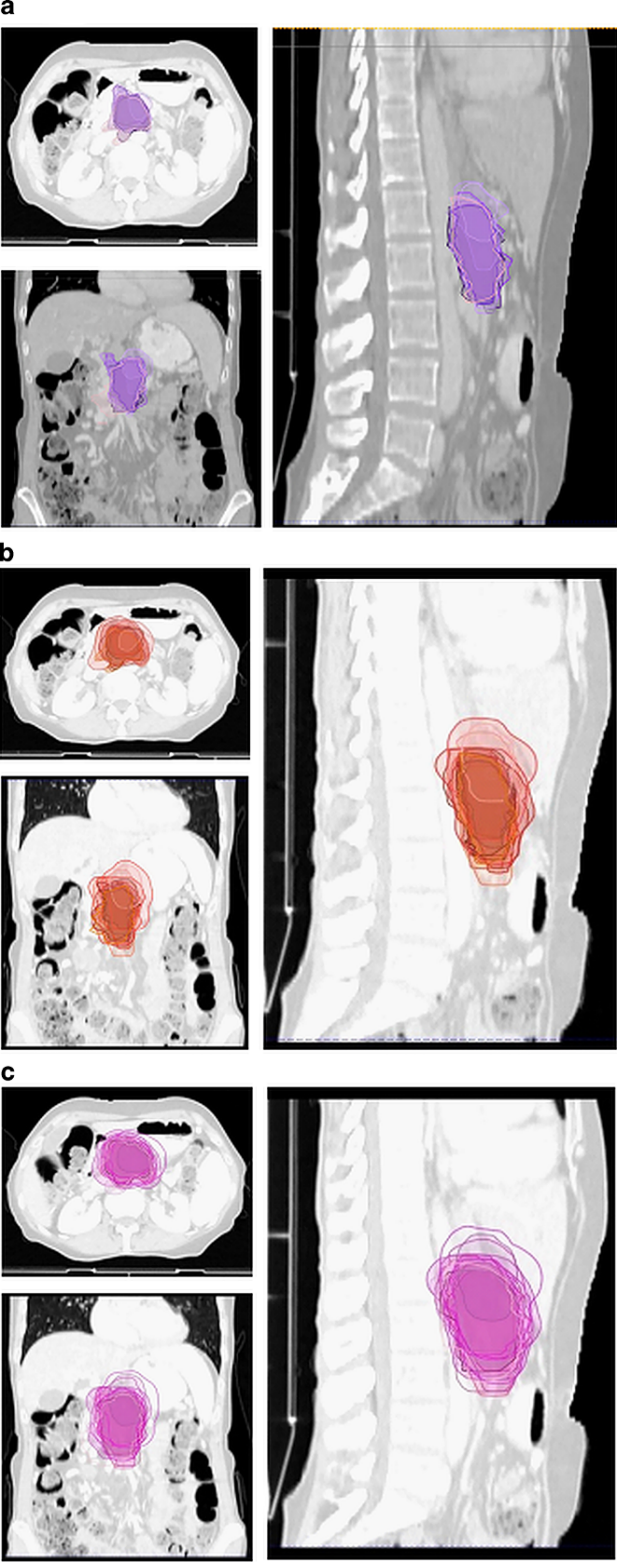
Case 3: patient with progression after initial chemotherapy. Definition of the target volumes performed based on 4D CT with IV contrast in combination with a diagnostic CT with IV contrast. **a** GTV, **b** ITV, **c** PTV

In the third case the patient was treated with SBRT due to progression of locally advanced pancreatic head carcinoma after initial chemotherapy and definition of the target volumes was based on 4D planning CT in treatment position with oral and intravenous (IV) contrast agent and an additional diagnostic MRI with IV contrast (Fig. 3). The median GTV was 45.7 (range 18.6–88.5) ml, the median ITV was 94.9 (range 32.9–237) ml, and the median PTV 158.35 (range 75–334.7) ml. For the GTVs the median DSC was 0.73, the median HD 21.61, the median PBD 0.38, and the median VS was 0.84, while for the ITVs the median DSC was 0.6, the median HD 23.64, the median PBD 0.66, and the median VS was 0.72.

## Discussion

Contouring of pancreatic tumors for SBRT is challenging for a number of reasons: 1) it is less frequently performed compared to, e.g., liver or lung lesions; 2) excellent imaging protocols are required for optimal visualization, including multimodal imaging, especially for pancreatic head lesions; 3) radiation oncologists require specific expertise, which may involve support by a radiologist. This study investigated interobserver variability in target volume delineation for three patients with pancreatic cancer, two with locally advanced disease and one with local relapse after resection, thus depicting typical SBRT indications. The large number of 24 participants with 19 structure sets per case allows us to obtain a relatively realistic impression of the variability of target volume definition in the expert community, showing a high agreement for the majority of the delineated volumes with median Dice coefficients > 0.7 for all GTVs.

To the best of our knowledge, this work represents the largest interobserver comparison of target volume delineations for SBRT in pancreatic cancer to date. A dedicated analysis of this topic in SBRT in patients with borderline resectable pancreatic cancer was published in 2017 by Holyoake [7], where eight radiation oncologists specializing in treating pancreatic cancer contoured one selected benchmark case for the SPARC trial. Contouring was based on contrast-enhanced CT and an FDG-PET/CT scan. In their analysis, the mean DSC for the entire GTV was 0.47 (0.38 to 0.56) and for the boost subvolume it was 0.61 (0.5 to 0.70). Additionally, this group reported the discordance indexes for GTV and boost volume, which were 0.65 and 0.39, respectively, i.e., better for the boost. The authors concluded that overcontouring of the GTV, which was larger than the gold standard in all participating centers, resulted in a significant risk for toxicity, especially duodenal bleeding. In another study, one borderline and one unresectable pancreatic cancer case were segmented with and without additional MRI information by 31 observers [8]. Additional FDG-PET information was tested in 25 pancreatic cancer patients manually segmented by two observers versus automated segmentation based on PET edges or SUV40% by Belli ML et al. [9]. Manual PET segmentation of pancreatic cancer resulted in a DSC of 0.73. However, there were only two observers and no comparison of PET- with CT-based segmentation was reported. Hence, the additional value of PET information to CT information is not clear. For conventionally fractionated radiotherapy, Fokas et al. [10] reported on one benchmark case which was contoured by 25 observers for the SCALOP trial. This group reported the Jaccard index (JCI), which is closely related to the DSC. The JCI was 0.57 for the GTV and 0.75 for the PTV. Interobserver agreement was better in our study compared to the benchmark case in SCALOP, assuming that DSC and JCI are interchangeable. As there was no ITV to be contoured in the SCALOP exercise, the PTV in that analysis resulted in a direct expansion of the GTV by 15 mm in all directions except for the craniocaudal direction, where the expansion was 20 mm. Better agreement of PTVs compared to GTVs was probably due to the absorption of undulating surfaces of the GTVs by the expansion margin used to create the PTVs.

This is the first study evaluating several metrics concerning interobserver variability and thus providing a more spherical approach. Recently, these metrics were reviewed for auto-segmentation and grouped into four domains: geometric, dosimetric, time-based, and qualitative [11]. Among these, only geometric and qualitative metrics are relevant for this study with a focus on geometric measures. Previous studies have considered a cut-off value of 0.7 for Jaccard/Dice metrics as a clinically adequate delineation [11]. Using this threshold, the maximal DSCs for all three GTVs were ≥ 0.85 and the median DSC of our analysis was above 0.7 for all three GTV datasets, and < 0.7 only for the ITV and the PTV in case 3, suggesting good overall quality. However, the minimal DSC values reveal that there is substantial variation in this data, with a large number of contours achieving a DSC < 0.7. Additionally, the use of arbitrarily determined cut-off values such as 0.7 for JCI or DSC should be questioned, because the definition of acceptable variations depends on the disease site [12] and the specific region of interest as well as weak correlations of geometric indices with dosimetric measures and physician ratings, as suggested recently [11]. Therefore, we have investigated additional parameters, namely the symmetric Hausdorff distance (HD), the probabilistic distance (PBD), and the volumetric similarity (VS). The combined used of all metrics reliably confirmed that in patient 3, the IOA was lesser compared to cases 1 and 2. We could show that when considering the different imaging scenarios for definition of the GTV, we observed better agreement concerning the overlap, alignment, and spatial distance in the first case using planning 4D CT without IV contrast with an additional planning PET/CT with abdominal compression and a diagnostic CT with IV contrast. Although in this case a diagnostic MRI was not performed, the agreement for the GTV definition was better compared to case 2 where both a diagnostic MRI and planning PET/CT were provided.

Additionally, we observed a difference in the definition of the GTV and ITV. While the IOA for the GTV was better in case 1, for the ITV and PTV, the IOA was better in case 2 using 4D PET/CT for planning. Especially concerning the VS, which is a volume-based metric, the use of 4D PET/CT was better than all other constellations for definition of all target volumes (GTV, ITV, PTV). On the contrary, the HD metric, which is a spatial distance metric used as a dissimilarity measure, was lower in case 1 using 4D CT without IV contrast and a PET/CT and diagnostic CT. In general, the use of FDG-PET/CT, either as 4D or as 3D, in treatment position with abdominal compression is superior for definition of the treatment volume, and especially the use of 4D planning PET/CT seems to improve IOA for the ITVs and PTVs. Thus, it should be considered as a useful imaging modality for target volume delineation in stereotactic radiotherapy of pancreatic cancer. However, according to the current ESTRO ACROP guideline, FDG-PET/CT is regarded as an optional additional imaging modality and a diagnostic CT, MRI, or FDG-PET/CT should be taken into consideration for target volume definition, but not a dedicated FDG-PET/CT scan in treatment position. [13].

Considering the relatively good agreement between observers, we hypothesize that for real-life conditions, SBRT delineation in pancreatic cancer is not the weakest link in the treatment chain and provides a good basis for treatment planning. We feel that treatment planning to reconcile adequate PTV coverage and protection of critical organs at risk has the potential to be more challenging. Therefore, we plan to use the herein presented three cases for a treatment planning study that will aim to analyze whether a large number of planners achieve comparable efficacy and safety.

Our study has a number of limitations such as the small number of patients. Additionally, one shortcoming of several evaluation metric results provided in the current literature is represented by changes as a function of the number of background voxels. In order to avoid this problem and to ensure fair comparisons, we only considered metrics that are not heavily affected by the number of background voxels.

## Conclusion

Interobserver variability was relatively low compared with other studies on delineation in pancreatic cancer in a large number of observers. Overall, this analysis suggests that the use of planning 3D PET/CT and preferably 4D PET/CT in treatment position with abdominal compression leads to better agreement using a variety of metrics and should be considered as an imaging modality for the definition of treatment volumes in pancreatic SBRT.
